# 超高效液相色谱-三重四极杆复合线性离子阱质谱法同时测定指印中36种降压药

**DOI:** 10.3724/SP.J.1123.2021.05012

**Published:** 2022-02-08

**Authors:** Qiuyao DU, Yunfeng ZHANG, Jifen WANG, Peng ZHAO, Xiaojun WU, Linpei DONG, Jiayi LI, Bingjie LIU

**Affiliations:** 1.公安部物证鉴定中心, 北京 100038; 1. Institute of Forensic Science, Ministry of Public Security, Beijing 100038, China; 2.中国人民公安大学侦查学院, 北京 100038; 2. School of Investigation, People’s Public Security University of China, Beijing 100038, China; 3.SCIEX亚太应用支持中心, 北京 100015; 3. SCIEX Asia Pacific Application Support Center, Beijing 100015, China

**Keywords:** 超高效液相色谱-三重四极杆复合线性离子阱质谱, 降压药, 指印, ultra performance liquid chromatography-triple quadrupole composite linear ion trap mass spectrometry (UPLC-Q-TRAP/MS), hypotensive drugs, fingerprints

## Abstract

指印中蕴含着供体摄入成分等相关信息,通过对其分析可对供体进行特征刻画,从而为案件侦查提供线索,指印分析也可用于药物摄入的定性检测,因此检验指印中的降压药具有重要的实际应用价值。建立了超高效液相色谱-三重四极杆复合线性离子阱质谱(UPLC-Q-TRAP/MS)同时检测指印中36种降压药的方法。前处理方法采用沉淀蛋白法,使用3×3 cm滤纸采集指印,将滤纸剪碎置于2 mL塑料离心管中,加入0.50 mL甲醇,涡旋混合1 min,超声振荡3 min,取出后以12000 r/min离心5 min,取上清液进样分析。采用ACQUITY UPLC^®^ BEH C18柱(100 mm×3.0 mm, 1.7 μm)分离,以0.01%甲酸水溶液和甲醇作为流动相进行梯度洗脱。质谱分析采用可编程多反应监测-信息关联采集-增强子离子(SMRM-IDA-EPI)扫描方式,在高灵敏度分析的同时进行二级谱库检索,增加定性结果的准确性。36种药物在0.05~50.00 ng/fingerprint范围内线性关系良好,相关系数(*r*)大于0.99,检出限和定量限分别为0.001~0.045 ng/fingerprint和0.002~0.050 ng/fingerprint,在0.25、2.50、25.00 ng/fingerprint 3个加标水平下的基质效应为79.0%~119.2%,回收率为79.3%~116.2%,日内精密度为0.2%~18.3%,日间精密度为1.6%~19.1%。使用该方法检测了87名高血压患者指印中的降压药,大部分样本中可检出其所服用的药物。该方法操作简单,灵敏度高,选择性好,适用于指印中降压药的筛查检验。

## Abstract (en)

Fingerprints contain important information such as the ingredients ingested by the donor. By analyzing the characteristic components in fingerprints, the donor can be characterized, which would provide insights for investigation of a given case. This approach can also be used in the qualitative monitoring of drug intake. Therefore, the examination of hypotensive drugs in fingerprints has significant value in practical application. This study established a method based on ultra performance liquid chromatography-triple quadrupole composite linear ion trap mass spectrometry (UPLC-Q-TRAP/MS) for the simultaneous determination of 36 hypotensive drugs in fingerprints. The pre-treatment method was based on protein precipitation. A 3×3 cm filter paper was cut into pieces and placed in a 2 mL plastic centrifuge tube after fingerprint collection. Then, 0.50 mL methanol was added, followed by vortex mixing for 1 min and ultrasonic oscillation for 3 min. The filter paper was centrifuged at 12000 r/min for 5 min, and the supernatant was withdrawn for sample analysis. An ACQUITY UPLC^®^ BEH C18 chromatographic column (100 mm×3.0 mm, 1.7 μm) was selected, with 0.01% aqueous formic acid and methanol as mobile phases for gradient elution. MS analysis involved scheduled multiple reaction monitoring-information dependent acquisition-enhanced product ion (SMRM-IDA-EPI) scanning. This method could be used to retrieve library researching during high-sensitivity analysis, which could increase the accuracy of qualitative results. The calibration curves showed good linearity in the range of 0.05-50.00 ng/fingerprint, with correlation coefficients (*r*) greater than 0.99 for all 36 analytes. The limits of detection and limits of quantification of the 36 hypotensive drugs were 0.001-0.045 ng/fingerprint and 0.002-0.050 ng/fingerprint, respectively. At spiked levels of 0.25, 2.50, 25.00 ng/fingerprint, the matrix effects, recoveries, intra-day precisions, and inter-day precisions of the 36 hypotensive drugs were 79.0%-119.2%, 79.3%-116.2%, 0.2%-18.3%, and 1.6%-19.1%, respectively. This method was used to detect hypotensive drugs in the fingerprints of 87 hypertensive patients, and hypotensive drug intakes were accurately detected in most cases. The established method is operationally simple, with high sensitivity and good selectivity, and it is suitable for screening and testing hypotensive drugs in fingerprints.

指印中包含多种内源性和外源性物质,如脂质、氨基酸、化妆品、药物、毒物、爆炸残留物等,其中蕴含着指印供体的生物特征、社会习性等相关信息^[1,2,3,4,5]^。通过对指印分析,可对供体进行特征刻画,从而为案件侦查提供线索。该方法在无法通过指印进行个体识别时有更加重要的意义^[6]^。另一方面,使用指印作为替代性生物基质进行药物检测是一个新的研究方向^[7]^。与典型的生物样品(例如血液和尿液)相比,指印采样和提取更加简单快捷,具有非侵入性,并且采样过程易于观察,难以掺假^[8,9]^。

高血压患者在我国人数多、分布广,近年来呈现年轻化趋势,且需要长期服药^[10]^。因此可通过检测指印中的降压药,获得供体服药及患病的相关信息,为侦查方向提供依据。此外,该方法还可用于定性药物监测或药物测定。降压药种类繁多,已有大量文献^[11]^报道了各类药物的作用机理、代谢规律和相互影响等,但指印中降压药的检验仍需要系统的研究。

检测指印中化学物质的方法包括光谱法^[12,13]^、免疫分析法^[14,15]^、色谱法^[16,17]^和质谱法^[18,19,20]^等。近年来,超高效液相色谱-串联质谱法(UPLC-MS/MS)由于其优越的灵敏度和选择性而成为各种基质中药物测定的首选技术^[21]^。但大多数UPLC-MS/MS使用的是三重四极杆(QQQ)或飞行时间(TOF)质谱,而使用三重四极杆复合线性离子阱质谱(Q-TRAP/MS)检测指印中药物的研究则鲜有报道^[22]^。UPLC-Q-TRAP/MS的可编程多反应监测-信息关联采集-增强子离子(SMRM-IDA-EPI)扫描方式可以在高灵敏度分析的同时获得化合物的二级质谱,因此其在同时筛查和定量分析方面具有优势^[23]^。

针对以上情况,本研究致力于结合UPLC-Q-TRAP/MS技术,建立指印中常见36种降压药的检验方法,探索指印化学分析用于药物测定的潜力,提升指印证据价值,为指印中药物检验在法庭科学中的应用提供技术支持。

## 1 实验部分

### 1.1 仪器、试剂与材料

LC分析使用LC-30A UPLC仪器(日本Shimadzu公司)进行,配备两个LC-30A泵、自动进样器、恒温柱室和脱气机。使用具有Turbo V^TM^离子源的5500 Q-TRAP/MS质谱系统(美国SCIEX公司)进行质谱分析。DHC-16500台式高速离心机(澳洲Kewlab公司); Vortex-Genie2可调速涡旋混合器(美国SI公司);超声振荡器(美国Cole-Parmer公司);电子分析天平(0.01 mg~220 g,德国Satorius公司)。

36种降压药对照品(见表1),纯度均大于97%,购自中国陶术、源叶、阿拉丁公司及中国食品药品检定研究院;甲醇、乙腈、甲酸、甲酸铵(均为HPLC级,美国Fisher Scientific公司);实验用水由Milli-Q去离子水系统(美国Millipore公司)制备。

**表 1:** 36种降压药的保留时间和质谱参数

No.	Compound	t_R_/min	Precursor ion/(m/z)	Product ions/(m/z)	CE/eV	DP/V
1	atenolol (阿替洛尔)	3.08	267.2	190.2, 145.1^*^	24, 32	80
2	hydrochlorothiazide (氢氯噻嗪)	3.19	296.0	268.9^*^, 205.0	-26, -30	-60
3	lisinopril (赖诺普利)	3.71	406.2	246.0, 84.2^*^	30, 29	80
4	triamterene (氨苯蝶啶)	3.77	254.2	237.2^*^, 104.1	34, 48	80
5	metoprolol (美托洛尔)	4.03	268.2	116.1^*^, 98.1	22, 24	80
6	captopril (卡托普利)	4.22	216.0	182.0^*^, 114.1	-15, -16	-50
7	chlortalidone (氯噻酮)	4.23	337.0	190.1^*^, 146.1	-22, -23	-80
8	bisoprolol (比索洛尔)	4.58	326.3	116.2^*^, 74.1	24, 28	80
9	olmesartan (奥美沙坦)	4.66	447.2	429.2, 207.1^*^	15, 26	60
10	propranolol (普萘洛尔)	4.81	260.2	183.1, 116.1^*^	23, 22	80
11	carvedilol (卡维地洛)	4.89	407.3	224.1^*^, 99.9	28, 32	80
12	enalapril (依那普利)	4.90	377.2	303.2, 234.2^*^	25, 24	80
13	furosemide (呋塞米)	4.90	329.0	285.0, 205.0^*^	-20, -28	-60
14	betaxolol (倍他洛尔)	4.92	308.3	116.1^*^, 98.0	25, 30	80
15	imidapril (咪达普利)	4.93	406.2	332.1, 234.2^*^	24, 25	80
16	eplerenone (依普利酮)	4.99	415.2	337.2, 163.0^*^	20, 20	80
17	verapamil (维拉帕米)	4.99	455.2	303.1, 165.0^*^	31, 34	80
18	nicardipine (尼卡地平)	5.05	480.3	315.2^*^, 166.0	28, 23	80
19	indapamide (吲达帕胺)	5.07	366.0	132.1^*^, 91.0	16, 38	22
20	perindopril (培哚普利)	5.14	369.3	172.1^*^, 98.1	26, 43	80
21	benidipine (贝尼地平)	5.16	506.3	315.2, 174.2^*^	31, 30	80
22	benazepril (贝那普利)	5.40	425.4	351.1^*^, 190.0	27, 39	80
23	ramipril (雷米普利)	5.52	417.2	234.2^*^, 130.1	25, 35	80
24	amlodipine (氨氯地平)	5.56	409.2	294.0, 238.0^*^	11, 11	80
25	telmisartan (替米沙坦)	5.59	515.4	497.2, 276.2^*^	45, 58	80
26	nifedipine (硝苯地平)	5.62	329.3	284.1^*^, 267.9	27, 30	50
27	candesartan (坎地沙坦)	5.71	441.2	423.2, 263.1^*^	14, 15	60
28	losartan (氯沙坦)	5.84	435.2	391.3, 157.1^*^	-18, -28	-60
29	lercanidipine (乐卡地平)	5.94	611.8	280.0^*^, 100.0	29, 50	80
30	irbesartan (厄贝沙坦)	5.97	427.2	399.3, 193.0^*^	-25, -32	-60
31	spironolactone (螺内酯)	6.02	417.3	341.3^*^, 106.9	13, 41	60
32	valsartan (缬沙坦)	6.13	433.9	350.1, 179.2^*^	-24, -29	-80
33	nitrendipine (尼群地平)	6.27	361.3	329.2, 315.2^*^	14, 15	80
34	felodipine (非洛地平)	6.96	382.1	236.1, 144.9^*^	-15, -16	-80
35	lacidipine (拉西地平)	7.30	473.4	354.1^*^, 310.1	17, 31	40
36	fosinopril (福辛普利)	7.51	564.4	436.3, 152.2^*^	20, 51	80

CE: collision energies; DP: declustering potential; * quantitative ion.

9 cm定性滤纸(杭州新华纸业公司);一次性氯化聚乙烯(CPE)手套(美国Ammex公司);移液器和2 mL离心管(德国Eppendorf公司)。

本研究通过了公安部物证鉴定中心科研伦理委员会的审查,并获得了志愿者的知情同意。实验所用的空白指印采集于4名健康志愿者:志愿者1,女,27岁;志愿者2,男,22岁;志愿者3,女,23岁;志愿者4,女,24岁。4名志愿者在实验期间均未服用任何药物,对其指印样品进行了36种降压药的筛查,结果均为阴性。

### 1.2 标准溶液的配制

准确称取10.00 mg标准品,分别用甲醇溶解定容至10.00 mL,得到质量浓度为1.00 mg/mL的单标准储备溶液。取适量每种单标准储备溶液,用甲醇稀释定容,得到质量浓度为10.00 μg/mL的混合标准溶液,于-20 ℃下避光储存。系列混合标准工作溶液使用甲醇连续稀释得到,现用现配。

### 1.3 样品的制备

使用3×3 cm的方形定性滤纸片采集指印,志愿者将手洗净晾干后戴一次性CPE手套2 min,将指头以中等力度捺印在承痕客体上,停留30 s,并标记捺印指印的位置。不同添加量的空白加标指印样品通过在空白指印区域加入25.00 μL适当浓度的混合标准溶液得到。

### 1.4 样品前处理

用剪刀将印有指印的滤纸剪碎,置于2 mL塑料离心管中,加入0.50 mL甲醇,涡旋混合1 min,超声振荡3 min,取出后以12000 r/min离心5 min,取上清液经0.22 μm有机膜过滤后进样分析。

### 1.5 实验条件

1.5.1 色谱条件

色谱柱:ACQUITY UPLC^®^ BEH C18柱(100 mm×3.0 mm, 1.7 μm),柱温:40 ℃,流动相:A相为0.01%甲酸水溶液,B相为甲醇,流速:0.4 mL/min。梯度洗脱程序:0~1.0 min, 5%B; 1.0~1.5 min, 5%B~35%B; 1.5~6.0 min, 35%B~95%B; 6.0~8.0 min, 95%B; 8.0~8.1 min, 95%B~5%B; 8.1~11.0 min, 5%B。进样量:5 μL。

1.5.2 质谱条件

离子源:电喷雾电离(ESI)源;离子源温度:550 ℃;扫描方式:正离子和负离子模式同时切换扫描;检测方式:SMRM-IDA-EPI模式;喷雾电压:5500 V(ESI^+^)/-4500 V(ESI^-^);雾化气压力:379 225 Pa;气帘气压力:241 325 Pa;辅助气压力:341 750 Pa;碰撞气压力:48 265 Pa;化合物射入电压和碰撞室射出电压在正离子模式下为10 V和17 V,在负离子模式下为-10 V和-15 V。其他质谱参数,包括保留时间、定量与定性离子对、去簇电压(DP)及碰撞能量(CE)见表1。仪器控制、数据采集和处理使用SCIEX OS 1.5软件。

## 2 结果与讨论

### 2.1 质谱条件的优化

查阅化合物的分子式,在电喷雾电离源,正、负离子模式下,先用针泵进样50.00 μg/L各目标化合物的单标准溶液,使用一级质谱全扫描(Q1 Scan)确定母离子的质荷比;使用二级离子扫描(Product Ion Scan),设定CE初始值为5 eV,以5 eV为步长自动调节CE,得到至少2~3个响应值高的特征子离子的质荷比,组建定量与定性分析离子对,优化CE和DP,以得到的参数初步建立多反应监测(MRM)质谱分析方法。优化结果见表1。

### 2.2 色谱条件的优化

液相色谱条件在实现良好的色谱行为方面起着关键作用^[24]^。实验选用ACQUITY UPLC^®^ BEH C18柱(100 mm×3.0 mm, 1.7 μm),实现了36种药物良好的分离效果。以ESI源为电离源的质谱分析需要对溶液中的样品进行电离,因此流动相的组成不仅会影响化合物的色谱峰形和保留时间,还会影响其离子化效率,进而影响检测灵敏度。实验比较了水和乙腈、水和甲醇、5 mmol/L甲酸铵水溶液和甲醇、0.01%甲酸水溶液和甲醇、0.1%甲酸水溶液和甲醇5种流动相组成。结果表明,使用乙腈作为有机相时化合物的分离效果和灵敏度没有使用甲醇时好;在流动相中加入甲酸铵后,样品中许多组分峰形较差且灵敏度较低;加入0.01%甲酸后分析灵敏度得到了提高,且分离效果和峰形较好;而加入0.1%甲酸时灵敏度下降。因此最终选择0.01%甲酸水溶液和甲醇作为流动相。

实验对梯度洗脱程序进行了优化,使用上述色谱条件36种化合物在11.0 min内获得了良好的色谱峰形和分离效果以及优异的灵敏度。以此色谱分析条件运行MRM方法,确定各待测化合物的保留时间,建立SMRM分析方法。SMRM是在MRM分析的基础上,根据目标化合物的保留时间设定窗口进行数据采集,在分析目标物数量较多的情况下,可以提高检测效率和灵敏度。36种药物的总离子流色谱图见图1。

**图1:**
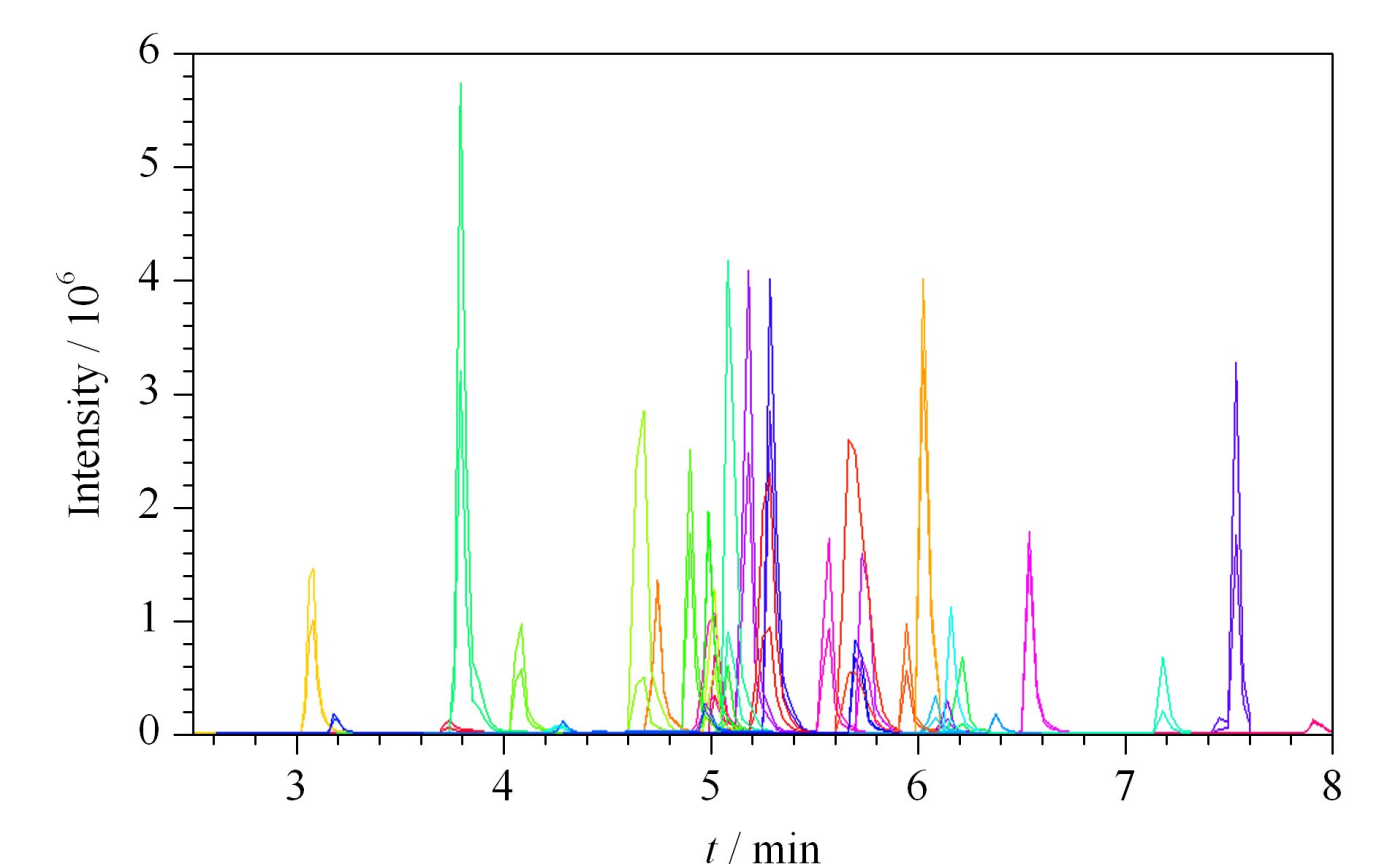
36种降压药的总离子流色谱图

### 2.3 EPI谱库的建立

UPLC-MS/MS常规的MRM检测方式,通过离子对的保留时间、质荷比、相对丰度比进行定性。本研究采用Q-TRAP/MS系统的SMRM-IDA-EPI扫描方式,在此基础上增加了二级谱库的匹配用于定性分析,可有效降低结果的假阳性率。该方法在SMRM分析的基础上,以预定义的信息关联采集标准自动触发增强子离子二级质谱扫描,其离子阱富集功能可获得高灵敏度的二级质谱信息^[25]^。分析在低(20 eV)、中(35 eV)、高(50 eV)3个碰撞能量下进行。对36种降压药的标准品溶液进行扫描,建立EPI二级谱库。将样品分析时获得的EPI二级谱图与谱库进行匹配,相似程度越高,定性结果的准确度越高。该方法通过一次进样同时满足了高灵敏度的定量分析和基于谱库的定性筛查需求。

### 2.4 前处理方法的优化

法庭科学毒物分析领域常用的生物检材前处理方法包括沉淀蛋白法、液液萃取、固相萃取、分散固相微萃取、低共熔溶剂萃取以及QuEChERS前处理方法等^[26]^。其中有机溶剂沉淀蛋白法具有操作简单快速、成本较低且不易引入影响检测的物质和破坏待测物结构等优点。考虑到指印中药物的含量较少,以及实际工作中快速高效的分析要求,本研究采用了操作较为简便的有机溶剂沉淀蛋白前处理方法。

使用滤纸采集的指印,采用直接剪取法进行提取,可减少提取过程中带来的损失,实际案件中由于客观因素无法进行剪取的,可以采用部分浸泡的方法进行提取。有机溶剂沉淀蛋白法最常用的沉淀剂是甲醇和乙腈。在添加水平为2.50 ng/fingerprint时,考察了0.50 mL甲醇、乙腈、50%甲醇水溶液作为沉淀剂时的提取效果。结果表明,甲醇和乙腈作为沉淀剂时的提取效果差别不大,且较50%甲醇水溶液作为沉淀剂时的提取效果好,由于甲醇毒性较乙腈低,因此选择使用甲醇作为沉淀剂。另外对比了使用0.50 mL和0.25 mL甲醇作为沉淀剂时的提取效果,沉淀剂用量为0.50 mL时提取效果较好,因而最终选用0.50 mL的甲醇作为沉淀剂。

### 2.5 方法学验证

2.5.1 线性范围、检出限和定量限

按照1.3节所述获得空白样品,并在空白指印区域添加适当浓度的混合标准溶液25.00 μL,得到添加量为0.05、0.25、0.50、1.25、2.50、5.00、12.50、25.00、50.00 ng/fingerprint的样品,按照前述实验条件和方法进行测定。以添加水平(*x*, ng/fingerprint)为横坐标、峰面积(*y*)为纵坐标进行线性回归。以离子对色谱峰信噪比(*S/N*)≥3和10时目标物的含量为方法的检出限(LOD)和定量限(LOQ)。实验结果表明,指印中36种药物在0.05~50.00 ng/fingerprint范围内线性关系良好,相关系数(*r*)大于0.99, LOD为0.001~0.045 ng/fingerprint, LOQ为0.002~0.050 ng/fingerprint,相关数据见表2。实验结果表明,该方法具有较高的灵敏度,可以满足实际测定的要求。

**表 2:** 36种降压药的回归方程、相关系数、检出限和定量限

Compound	Regression equation	r	LOD/(ng/fingerprint)	LOQ/(ng/fingerprint)
Atenolol	y=9.05×10^4^ x+2.50×10^4^	0.9950	0.014	0.048
Hydrochlorothiazide	y=4.05×10^4^ x+3.15×10^3^	0.9993	0.001	0.003
Lisinopril	y=1.26×10^4^ x+2.24×10^4^	0.9958	0.045	0.050
Triamterene	y=5.39×10^5^ x+5.69×10^3^	0.9958	0.001	0.004
Metoprolol	y=1.48×10^5^ x+2.56×10^4^	0.9979	0.003	0.011
Captopril	y=1.06×10^4^ x+1.70×10^4^	0.9903	0.035	0.050
Chlortalidone	y=3.62×10^4^ x+4.49×10^3^	0.9979	0.007	0.022
Bisoprolol	y=2.23×10^5^ x+6.91×10^4^	0.9982	0.001	0.002
Olmesartan	y=2.95×10^5^ x+4.33×10^4^	0.9988	0.004	0.014
Propranolol	y=3.27×10^5^ x+1.61×10^5^	0.9960	0.001	0.005
Carvedilol	y=3.60×10^5^ x+1.54×10^5^	0.9964	0.002	0.007
Enalapril	y=1.89×10^5^ x+3.08×10^4^	0.9992	0.001	0.007
Furosemide	y=8.57×10^4^ x+1.54×10^4^	0.9982	0.026	0.050
Betaxolol	y=1.67×10^5^ x+7.48×10^4^	0.9970	0.002	0.007
Imidapril	y=1.14×10^5^ x+1.29×10^4^	0.9987	0.003	0.011
Eplerenone	y=3.96×10^4^ x+4.69×10^3^	0.9996	0.004	0.013
Verapamil	y=2.12×10^5^ x+5.59×10^4^	0.9980	0.001	0.003
Nicardipine	y=5.07×10^5^ x+3.14×10^5^	0.9948	0.001	0.003
Indapamide	y=6.05×10^4^ x+9.59×10^3^	0.9988	0.003	0.010
Perindopril	y=4.99×10^5^ x+1.40×10^5^	0.9983	0.001	0.003
Benidipine	y=4.97×10^5^ x+2.41×10^5^	0.9964	0.001	0.005
Benazepril	y=3.09×10^5^ x+3.52×10^4^	0.9990	0.002	0.007
Ramipril	y=3.03×10^5^ x+7.46×10^4^	0.9986	0.001	0.004
Amlodipine	y=1.96×10^5^ x+1.79×10^4^	0.9987	0.007	0.022
Telmisartan	y=6.30×10^5^ x+4.02×10^5^	0.9952	0.002	0.005
Nifedipine	y=2.05×10^5^ x+1.68×10^4^	0.9976	0.001	0.005
Candesartan	y=1.33×10^5^ x+6.37×10^3^	0.9993	0.002	0.007
Losartan	y=1.39×10^5^ x+5.19×10^3^	0.9989	0.009	0.030
Lercanidipine	y=8.45×10^4^ x+3.97×10^4^	0.9967	0.004	0.014
Irbesartan	y=3.29×10^4^ x+8.70×10^3^	0.9984	0.008	0.027
Spironolactone	y=1.16×10^5^ x+4.92×10^4^	0.9993	0.005	0.018
Valsartan	y=5.08×10^4^ x+3.65×10^3^	0.9992	0.005	0.018
Nitrendipine	y=3.38×10^5^ x+1.27×10^5^	0.9976	0.006	0.019
Felodipine	y=4.77×10^4^ x+1.13×10^4^	0.9960	0.003	0.009
Lacidipine	y=2.24×10^5^ x+3.71×10^4^	0.9937	0.001	0.004
Fosinopril	y=3.12×10^4^ x+6.59×10^3^	0.9902	0.005	0.017

*y*: peak area; *x*: content, ng/fingerprint.

2.5.2 基质效应

指印中存在胆固醇、脂肪酸、氨基酸、角鲨烯等复杂物质,因此需要考察基质效应(ME),以确保检测的准确性。按照1.4节中所述前处理方法取空白指印进行甲醇沉淀蛋白的操作步骤,取离心后的上清液作为空白基质溶液,用空白基质溶液配制混合标准溶液进样分析,得到目标物的峰面积(*A*),同时测定相同浓度混合标准溶液中目标物的峰面积(*B*),根据ME=*A/B*×100%计算基质效应。分别计算药物添加水平为0.25、2.50、25.00 ng/fingerprint时的基质效应,每个加标水平平行测定5次,实验数据见表3。当ME<100%时,说明基质对目标物有抑制作用,当ME>100%时,则存在基质增强效应。实验结果表明,指印中所有目标药物的基质效应为79.0%~119.2%,说明本研究采用的方法基质效应较弱,定量分析结果具有准确性和可靠性。

**表 3:** 36种降压药的基质效应和加标回收率(*n*=5)

Compound	Matrix effects/%				Recoveries/%				Intra-day RSDs/%				Inter-day RSDs/%		
	0.25 ng/fp	2.50 ng/fp	25.00 ng/fp		0.25 ng/fp	2.50 ng/fp	25.00 ng/fp		0.25 ng/fp	2.50 ng/fp	25.00 ng/fp		0.25 ng/fp	2.50 ng/fp	25.00 ng/fp
Atenolol	119.2	106.6	105.4		84.7	91.5	82.9		18.0	13.0	3.7		17.4	13.9	12.5
Hydrochlorothiazide	81.5	83.8	86.8		95.8	83.7	83.3		4.4	4.1	0.6		5.1	3.5	2.5
Lisinopril	114.1	114.4	115.1		81.4	81.4	80.5		5.2	2.7	3.6		6.2	3.2	4.3
Triamterene	99.5	95.5	94.7		80.7	81.8	79.3		10.2	9.6	9.6		9.6	8.5	9.1
Metoprolol	93.0	96.0	93.1		97.8	106.1	92.3		2.7	3.6	5.3		3.5	3.1	5.7
Captopril	81.9	82.3	85.7		80.1	114.3	83.5		18.3	15.6	13.6		14.6	14.2	12.7
Chlortalidone	87.1	98.3	98.7		85.4	97.8	95.8		1.7	4.9	6.4		2.9	3.7	5.0
Bisoprolol	92.2	93.5	99.8		94.8	99.9	100.0		1.4	1.6	2.1		3.6	2.3	2.2
Olmesartan	92.3	114.2	95.9		84.9	98.6	94.6		1.5	3.2	2.9		2.9	3.9	3.5
Propranolol	88.6	99.2	96.0		92.2	106.9	89.9		2.0	4.9	5.5		3.2	3.4	4.5
Carvedilol	90.5	96.5	100.9		83.1	83.1	80.9		5.5	5.0	4.0		7.5	4.9	6.2
Enalapril	90.5	90.7	101.1		88.1	100.4	95.4		4.8	2.1	2.2		4.8	4.3	2.2
Furosemide	97.0	93.5	105.6		82.2	81.3	83.0		10.4	10.6	4.0		8.8	8.4	3.5
Betaxolol	95.2	87.0	95.8		87.9	107.2	98.6		1.9	1.6	3.4		4.1	3.3	4.9
Imidapril	88.8	95.7	95.4		83.2	93.0	89.4		5.5	3.6	3.9		4.3	3.7	5.1
Eplerenone	97.4	86.0	100.5		84.9	96.6	93.2		1.4	4.0	1.0		3.1	4.1	4.1
Verapamil	95.0	94.8	94.2		98.9	98.3	102.0		2.3	3.7	1.1		3.0	3.0	5.7
Nicardipine	93.3	97.8	82.0		84.8	95.2	92.1		2.3	1.8	2.7		3.1	2.6	4.1
Indapamide	95.2	99.8	94.4		83.0	85.4	100.1		1.4	0.8	2.3		4.5	4.2	4.2
Perindopril	94.8	94.1	99.3		86.9	101.3	100.6		2.6	2.8	1.4		3.3	3.4	2.1
Benidipine	91.3	96.4	92.0		80.5	102.9	100.3		2.0	3.5	2.7		1.6	3.2	3.2
Benazepril	90.9	90.4	103.9		89.0	81.7	87.9		3.0	4.3	2.7		3.0	4.9	2.7
Ramipril	90.5	94.6	100.6		96.3	98.9	97.9		2.2	3.1	1.9		2.8	4.9	3.0
Amlodipine	94.1	87.8	93.9		79.4	92.9	84.3		5.3	1.0	5.0		6.0	4.1	5.2
Telmisartan	89.2	88.7	98.4		91.0	102.6	94.7		0.2	3.2	2.2		6.1	5.1	3.7
Nifedipine	82.7	82.5	93.9		80.4	84.3	81.1		9.5	5.2	2.8		7.0	7.5	4.6
Candesartan	87.6	113.5	95.4		116.2	94.1	92.6		5.0	6.9	1.7		7.5	7.5	3.7
Losartan	83.9	97.4	113.7		79.3	93.8	96.3		5.1	6.3	3.7		4.6	5.4	4.7
Lercanidipine	90.9	81.6	87.1		86.7	111.1	104.0		5.1	3.4	5.2		7.3	7.7	5.7
Irbesartan	85.0	88.9	104.1		93.6	96.7	100.9		1.7	1.0	1.5		3.2	4.0	4.7
Spironolactone	102.9	104.1	109.1		110.7	97.5	95.5		12.7	6.5	9.7		13.4	19.1	13.5
Valsartan	79.0	114.4	105.4		103.5	113.3	102.1		4.3	8.9	1.2		5.8	7.7	3.5
Nitrendipine	92.0	86.2	95.2		85.1	94.9	97.7		5.6	5.3	5.9		10.1	9.4	6.5
Felodipine	104.6	100.8	98.4		80.9	97.1	84.4		1.2	10.0	5.5		5.7	8.9	7.8
Lacidipine	81.7	85.0	100.9		80.6	86.5	91.8		6.1	7.0	9.9		8.6	4.4	7.4
Fosinopril	103.8	105.1	106.5		105.0	87.5	82.2		14.0	11.2	13.9		15.5	17.0	12.5

ng/fp: ng/fingerprint.

2.5.3 回收率和精密度

在空白指印中分别添加0.25、2.50和25.00 ng/fingerprint的混合标准溶液,按照上述前处理方法进行回收试验,得到目标物的峰面积(*C*),根据回收率=*C/A*×100%进行计算,每个加标水平在1 d内平行测定5次,回收率数据见表3。计算相对标准偏差,得到日内精密度(intra-day RSD),连续进样5 d,计算日间精密度(inter-day RSD),相关实验数据见表3。实验数据表明,指印中36种药物的回收率为79.3%~116.2%,日内精密度为0.2%~18.3%,日间精密度为1.6%~19.1%,说明该方法的重复性和准确性良好,证明了其在指印中降压药分析中的可靠性和实用性。

### 2.6 实际样品的测定

本研究共招募87名患有高血压并服用降压药的志愿者,其中男性40人,女性47人,涵盖不同年龄段、不同服药时间和不同服药量,样本具有一定的代表性。志愿者将手洗净、晾干后戴一次性CPE手套2 min,将指头以中等力度捺印在剪裁好的滤纸上,停留30 s,使指印汗液转移到承痕客体上,保证成功采样。每个指印样品严格按照相同的方法采集,保证样品的稳定性。按照本文建立的方法对样品进行提取分析,每个样品平行测定3次。通过待测物离子对的质荷比、保留时间、相对丰度比以及EPI谱库的匹配情况进行定性分析。志愿者服用的药物、服药人数及指印中检出其所服用药物的信息见表4(部分志愿者同时服用2种降压药)。根据表中数据,指印中降压药的检出率可达77%以上。对于未检出的样品,可能与指印中的汗液量和受试者的药代动力学差异有关^[27]^。实验结果表明,药物的排泄可通过皮肤的汗腺和/或皮脂腺发生,本研究建立的方法适用于指印中降压药的检验。

**表 4:** 志愿者服用药物、服药人数和指印中药物检出人数

Hypotensive drug	Number of people taking drugs	Number of people detected drugs from fingerprints	Hypotensive drug	Number of people taking drugs	Number of people detected drugs from fingerprints
Amlodipine	26	22	Irbesartan	3	2
Felodipine	1	1	Losartan	1	1
Nifedipine	34	26	Telmisartan	10	7
Benazepril	2	2	Valsartan	2	1
Captopril	4	3	Bisoprolol	1	1
Enalapril	3	2	Metoprolol	5	4
Candesartan	2	1	Propranolol	2	2
Irbesartan	3	2	Hydrochlorothiazide	7	5
Losartan	1	1	Indapamide	4	3

## 3 结论

本研究建立了UPLC-Q-TRAP/MS检测指印中36种降压药的方法。该方法操作简单,分析速度快,具有较高的灵敏度和选择性。这种方法通过对指印进行化学分析来测定药物,具有非侵入性的优点,且样品收集及制备程序简单,是可用于药物监测的新方法。该方法还进一步增强了对指印供体进行特征刻画的研究,为指印中药物检测在法庭科学中的应用及现场痕量证据的深度挖掘提供了技术支持。
